# Exercise Stress Echocardiography–Guided Balloon Pulmonary Valvuloplasty for Pulmonary Valve Stenosis With a Moderate Pressure Gradient and Exercise Intolerance: A Case Report

**DOI:** 10.1155/cric/8011634

**Published:** 2026-08-03

**Authors:** Soichi Komaki, Yoshihiko Kodama, Kohei Kurogi, Kinuko Yamamoto, Takeshi Ideguchi, Yunosuke Matsuura, Hiroshi Moritake, Koichi Kaikita

**Affiliations:** ^1^ Division of Cardiovascular Medicine and Nephrology, Department of Internal Medicine, Faculty of Medicine, University of Miyazaki, Miyazaki, Japan, miyazaki-u.ac.jp; ^2^ Division of Pediatrics, Faculty of Medicine, University of Miyazaki, Miyazaki, Japan, miyazaki-u.ac.jp

**Keywords:** balloon pulmonary valvuloplasty, cardiopulmonary exercise testing, exercise stress echocardiography, pulmonary valve stenosis

## Abstract

Current guidelines recommend balloon pulmonary valvuloplasty (BPV) for symptomatic adults with moderate or severe pulmonary valve stenosis (PVS) when the valve anatomy is suitable. However, when the resting gradient is near the lower boundary of the moderate range, establishing whether exercise intolerance is attributable to PVS can be challenging. We describe the case of a 52‐year‐old woman with longstanding PVS who developed progressive exercise intolerance. Resting echocardiography revealed a peak gradient of 38 mmHg, which remained stable during follow‐up. Exercise stress echocardiography (ESE) demonstrated a blunted stroke volume response, whereas cardiopulmonary exercise testing (CPET) showed reduced peak VO_2_. Based on these objective findings, BPV was performed for symptomatic moderate PVS. Following successful intervention, both the stroke volume response and peak VO_2_ improved markedly. This case highlights that exercise‐based diagnostic modalities, particularly ESE in conjunction with CPET, can help guide treatment decisions in symptomatic moderate PVS.

## 1. Introduction

Pulmonary valve stenosis (PVS) is a congenital cardiac anomaly most often diagnosed in childhood. Current guidelines recommend intervention for symptomatic adults with moderate or severe valvular PVS when the valve anatomy is suitable [1, 2]. However, in patients with exercise intolerance and a resting pressure gradient at the lower end of the moderate range, attributing symptoms to PVS and determining whether and when to intervene can be challenging [3]. In such cases, exercise‐based testing may provide important diagnostic insights; however, few studies have evaluated the utility of exercise stress echocardiography (ESE) or cardiopulmonary exercise testing (CPET) in patients with PVS. We present a case in which exercise‐based assessment, particularly ESE, played an important role in guiding the management of symptomatic moderate PVS.

## 2. Case Presentation

**Figure 1:**
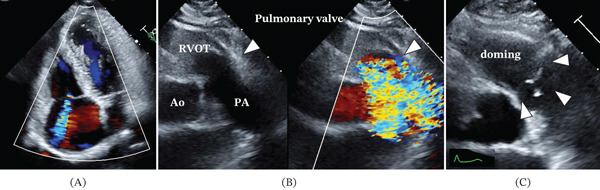
Echocardiographic findings at rest. (A) Apical four‐chamber view showing right atrial enlargement. (B) Parasternal short‐axis view demonstrating turbulent flow across the pulmonary valve. (C) Systolic dome‐shaped morphology of the pulmonary valve consistent with valvular stenosis.

A 52‐year‐old woman was diagnosed with PVS in childhood and was followed conservatively due to the absence of symptoms. During long‐term follow‐up, the peak transvalvular pressure gradient remained approximately 35 mmHg, with preserved right ventricular (RV) function, as indicated by a tricuspid annular plane systolic excursion (TAPSE) of 25 mm and a right ventricular fractional area change (RV‐FAC) of approximately 60%. At 50 years of age, she developed exertional fatigue during brisk walking, although its relationship to PVS was uncertain. Resting echocardiography showed preserved biventricular systolic and diastolic function, a peak transvalvular pressure gradient of 38 mmHg, a TAPSE of 21 mm, an RV free wall longitudinal strain of −31.9%, an RV‐FAC of 49.1%, right atrial enlargement, and a dome‐shaped pulmonary valve with turbulent flow consistent with valvular stenosis **(**Table 1 and Figure 1 **)**. Blood tests showed a B‐type natriuretic peptide level of 10.0 pg/mL and a troponin T level of 0.004 ng/mL.

**Table 1:** Clinical and functional assessments before and 1 month after balloon pulmonary valvuloplasty (BPV).

Parameter	Before BPV	1 month after BPV
**Clinical status and cardiac biomarkers**		
NYHA class	II	I
BNP (pg/mL)	10.0	6.4
cTnT (ng/mL)	0.004	0.004
**Resting echocardiography**		
PV morphology	Mild thickening, doming	Mild thickening
TAPSE (mm)	21	24
RV FWLS (%)	−31.9	−28.9
RV‐FAC (%)	49.1	63.3
PVA (cm^2^)	0.86	1.32
**ESE (rest)**		
HR (bpm)	77	64
SV (mL)	65.5	78.2
CO (L/min)	5.0	5.0
Vmax (m/s)	3.2	2.6
**ESE (peak)**		
HR (bpm)	128	126
SV (mL)	51.0	81.9
CO (L/min)	6.5	10.3
Vmax (m/s)	4.1	3.7
**CPET**		
Workload (W)	94	96
Peak HR (bpm)	127	126
Peak VO_2_ (mL/kg/min) (% predicted)	18.8 (79%)	21.9 (92%)
VT (mL/kg/min) (% predicted)	10.1 (63%)	15.8 (98%)

Abbreviations: BNP, B‐type natriuretic peptide; BPV, balloon pulmonary valvuloplasty; CO, cardiac output; CPET, cardiopulmonary exercise testing; cTnT, cardiac troponin T; ESE, exercise stress echocardiography; HR, heart rate; PV, pulmonary valve; PVA, pulmonary valve area; RV‐FAC, right ventricular fractional area change; RV FWLS, right ventricular free wall longitudinal strain; SV, stroke volume; TAPSE, tricuspid annular plane systolic excursion; Vmax, peak transvalvular velocity; VT, ventilatory threshold.

**Figure 2:**
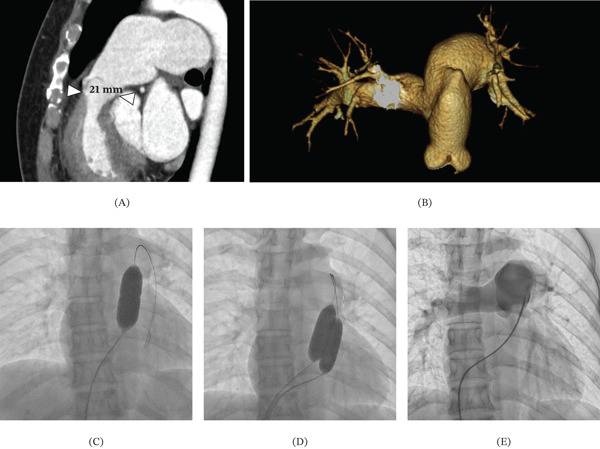
Preprocedural imaging and interventional procedure. (A) Contrast‐enhanced computed tomography acquired during systole demonstrated a 21‐mm pulmonary valve annulus (white arrowheads), without calcification or evidence of supravalvular or subvalvular stenosis or focal narrowing of the right ventricular outflow tract. (B) Post‐stenotic dilatation of the main pulmonary artery. (C) Initial balloon pulmonary valvuloplasty using a single 22‐mm balloon (2 atm), which resulted in a residual pressure gradient. (D) Subsequent double‐balloon dilation with two 16‐mm balloons inflated simultaneously (2 atm each), achieving more effective valvular expansion and a substantial reduction in the pressure gradient. (E) Final pulmonary angiography showing only mild pulmonary regurgitation.

To assess exercise tolerance and hemodynamic response, ESE using a bicycle ergometer was performed under continuous electrocardiographic (ECG) and blood pressure monitoring. ESE was conducted after confirming that the patient was clinically stable, with no history of arrhythmias, syncope, or cyanosis. During the test, stroke volume declined from 65.5 mL at rest to 51.0 mL at 75 W, cardiac output showed only a modest increase from 5.0 to 6.5 L/min (Table 1), and the tricuspid regurgitation pressure gradient rose from 31 to 100 mmHg. No adverse events occurred during or after the test. CPET revealed a peak VO_2_ of 18.8 mL/kg/min, confirming impaired exercise tolerance. Computed tomography showed no obstruction within the RV outflow tract or pulmonary artery. These findings provided objective evidence that the patient′s exercise intolerance was attributable to moderate PVS, and balloon pulmonary valvuloplasty (BPV) was performed **(**Figure 2 **)**. The procedure reduced the catheter‐derived peak‐to‐peak pressure gradient from 42 to 19 mmHg. Compared with the pre‐BPV assessment, ESE performed at the 1‐month follow‐up demonstrated improved stroke volume and cardiac output responses, with the values at 75 W increasing from 51.0 to 81.9 mL and from 6.5 to 10.3 L/min, respectively. Additionally, CPET demonstrated an increase in peak VO_2_ from 18.8 to 21.9 mL/kg/min **(**Table 1 **)**.

## 3. Discussion

Although BPV is well established as the standard therapy for severe PVS, with excellent long‐term outcomes in both pediatric and adult populations, its role in patients with moderate PVS and reduced exercise tolerance remains less clearly defined. Clinical decision‐making relies largely on symptoms and resting echocardiographic assessment, but this approach may fail to adequately capture the functional impact of the lesion [4]. This case underscores the limitations of resting parameters, which may underestimate the true hemodynamic burden of moderate PVS, particularly in symptomatic patients.

In this case, functional capacity improved markedly following BPV, as demonstrated by increases in cardiac output and peak VO_2_ during exercise testing. These findings emphasize the diagnostic value of dynamic assessment. ESE and CPET can unmask hemodynamic abnormalities not evident at rest, thereby providing valuable clinical guidance in patients with borderline resting pressure gradients [5, 6]. Although such approaches are well recognized in the management of aortic stenosis and mitral regurgitation, there are very few published reports describing their application in PVS. Given the limited evidence available, further studies are needed to clarify the clinical role of exercise‐based assessment in PVS.

In valvular heart disease more broadly, objective abnormalities during exercise may precede clinically apparent symptoms and provide prognostic information [7]. In PVS, an attenuated stroke volume response during exercise, despite preserved RV function at rest, may reflect limited RV contractile reserve resulting from chronic pressure overload [8–11]. Thus, when the resting pressure gradient is relatively modest despite the presence of symptoms, an impaired ability of the RV to augment stroke volume and cardiac output in response to an exercise‐induced increase in afterload may provide a plausible physiological explanation for exercise intolerance. RV maladaptation to long‐standing pressure overload has been recognized as an important determinant of prognosis in congenital heart disease and pulmonary hypertension [12, 13].

Importantly, an unexpected discrepancy between the pressure gradient across the pulmonary valve and the RV systolic pressure estimated from the tricuspid regurgitant jet should prompt careful reassessment of RV anatomy, loading conditions, measurement accuracy, and other potential confounders. In this case, multimodality imaging showed no evidence of intraventricular, subvalvular, or supravalvular obstruction. These findings highlight the importance of integrating imaging findings with invasive and exercise hemodynamic data when considering intervention for PVS, particularly in symptomatic patients with moderate resting gradients.

The postintervention improvement in peak VO_2_ supports the concept that exercise‐based testing can detect clinically relevant functional impairment and provide objective justification for intervention in moderate PVS. By demonstrating a physiological link between exercise intolerance and moderate PVS, this case provides clinically relevant insight into an underexplored aspect of valvular heart disease management.

Taken together, these observations highlight the importance of incorporating exercise‐based approaches into the assessment of symptomatic moderate PVS. This case also emphasizes the value of comprehensive multimodality assessment in guiding management decisions. By providing objective evidence of exercise intolerance and unmasking limited RV functional reserve, these tools may improve risk stratification and help identify patients who could benefit from intervention.

## 4. Conclusion

This case demonstrates that exercise‐based diagnostic approaches, particularly ESE, can provide important information for treatment decision‐making in moderate PVS. CPET can provide complementary confirmation of limited exercise capacity. These tools may help identify symptomatic patients with borderline resting pressure gradients who could benefit from BPV. Given the paucity of published reports on exercise‐based assessment in PVS, further studies are warranted to validate these approaches and define their role in refining treatment strategies.

## Author Contributions

Soichi Komaki, Yoshihiko Kodama, and Kohei Kurogi were responsible for the clinical care of the patient and prepared the initial draft of the manuscript. Yoshihiko Kodama and Yunosuke Matsuura contributed to revising and editing the manuscript. Kinuko Yamamoto, Takeshi Ideguchi, Hiroshi Moritake, and Koichi Kaikita provided important clinical insights that improved the manuscript.

## Funding

No funding was received for this manuscript.

## Disclosure

The authors have nothing to report.

## Ethics Statement

Ethical approval was not required for this single‐patient case report in accordance with institutional policy.

## Consent

Written informed consent was obtained from the patient for publication of this case report and any accompanying images.

## Conflicts of Interest

The authors declare no conflicts of interest.

## Data Availability

All data used to support the findings of this study are available from the corresponding author upon reasonable requests.
